# Enlarged Tonsils, What Shall We Do for Them?

**Published:** 1889-01

**Authors:** 


### ENLARGED TONSILS—WHAT SHALL WE DO WITH THEM.

Dr. Shields, at Med. Socity of Va. said: He had never seen a single case of true hypertrophied tonsils permanently removed by the application of any of toe astringents, absorbents or mild caustics suggested, or by the injection into their substance of iodine, ergotin, etc. In soft varieties or in the case of young children where prompt contraction of the blood vessels follows cutting, he uses the tonsillotome; but in the hard fibrous varieties and in adults, where there is danger of alarming hemorrhage he prefers the use of the galvano-cautery.

From six to eight points over the surface of the tonsil should be touched with the moxa point electrode heated to a red heat at one sitting, and usually from four to eight sittings are required to shrink up the tonsil thoroughly. If a 10 per cent. solution of cocaine is firs applied, patients will usually complain of no pain. Dr. Shielc's conclusions were: In all cases except young children or where the tonsil is very soft, the galvanocautery method of removal is to be preferred, because, 1st. The tonsil can be more nearly restored to its normal proportions. 2nd. Irregularly shaped masses of hypertrophied tissue can be removed that could not be encircled by the tonsillotome. 3rd. She effect seems to be more permanent. 4th. It is devoid of all danger.

